# Editorial: Translational opportunities for AI in glaucoma

**DOI:** 10.3389/fopht.2023.1299582

**Published:** 2023-11-06

**Authors:** Kevin C. Chan, Rebecca M. Sappington

**Affiliations:** ^1^ Department of Ophthalmology, New York University Grossman School of Medicine, NYU Langone Health, New York University, New York, NY, United States; ^2^ Department of Radiology, New York University Grossman School of Medicine, NYU Langone Health, New York University, New York, NY, United States; ^3^ Department of Biomedical Engineering, Tandon School of Engineering, New York University, New York, NY, United States; ^4^ Department of Biochemistry, Wake Forest University School of Medicine, Winston-Salem, NC, United States; ^5^ Department of Ophthalmology, Wake Forest University School of Medicine, Winston-Salem, NC, United States

**Keywords:** artificial intelligence, ChatGPT, glaucoma, intraocular pressure, optical coherence tomography (OCT), teleglaucoma, translational research

Recent advancements in artificial intelligence (AI), machine learning, and deep learning have revolutionized medical imaging and clinical assessment in ophthalmic studies, and continue to provide novel insights into the origins and management of various ocular diseases and disorders. Despite the rapid growth of this field, in multifactorial diseases such as glaucoma, the use of AI protocols for disease detection and clinical management is challenged by the diversity in patient populations, variability between imaging platforms, and access to imaging modalities and healthcare in developing countries. In the preclinical space, development of AI approaches for determining glaucoma pathology provides a unique opportunity to address some of these clinical challenges by building mathematical and computational models via well-controlled experimental conditions and simplified protocols. This Research Topic, *Translational Opportunities in Glaucoma*, presents 9 articles from 12 countries that illustrate current efforts to derive novel AI-based approaches and identify areas for critical refinements for AI use in glaucoma, so as to enable broad applications for basic science research, clinical translations, and glaucoma care.

## Refinement and evolution of AI in glaucoma

The perspective article by Ma et al. highlights the opportunity for translational advancement in glaucoma via integration of AI approaches in preclinical models and clinical populations. This article discusses recent progress, opportunities, and challenges for the application of AI to scientific discoveries and emphasizes the potential for a paradigm of reverse translation, where clinical data support patient-centered hypothesis generation that is followed by transitioning into basic science studies for hypothesis validation. To facilitate actionable progress in this area, the authors identified specific research opportunities for reverse translation of AI in glaucoma, including disease risk and progression prediction, pathology characterization, and sub-phenotype identification.

The systematic review by Thompson et al. highlights innovative advances and improvements in deep learning algorithms for automated detection of glaucomatous damage and progression on optical coherence tomography (OCT) imaging over the past decade. Current efforts to translate these AI algorithms to detect glaucomatous damage on fundus photography are also reviewed, whereas key limitations including demographic representation, ground truth references, and registration are identified. Based on these limitations, this review discusses the prospects of using preclinical models of aging and eye disease to inform AI refinement in clinical populations, along with the potential use of existing clinical datasets and algorithms to advance preclinical translation.

## AI opportunities for direction of care in glaucoma

The original research by Al Dalgan et al. used a mixed linear effects model to evaluate the relationship between variations in intraocular pressure (IOP) and changes in axial length for pediatric patients with primary congenital glaucoma. The study focused on post-surgical outcomes in 72 patients for a period of 4 years. The study concluded that substantial reductions in IOP were required for at least 3 months to regress IOP-dependent changes in axial length. These findings provide concrete metrics for surgical outcomes related to both IOP and axial length that could be incorporated in an AI approach to manage and predict treatment efficacy in primary congenital glaucoma.

In a case report by Chen et al., magnetic resonance imaging (MRI) and computed tomography (CT) revealed that a presumed case of acute angle closure glaucoma was a secondary glaucoma associated with choroidal metastatic carcinoma and Sjögren syndrome. This study highlights an opportunity for cross-domain and cross-modality AI models to identify aspects of atypical presentation that could recommend alternative diagnostic protocols.

The review article by Aktas and Ikiz summarizes current surgical techniques for the management of pediatric glaucoma and proposes minimally invasive glaucoma surgery (MIGS) as an advantageous approach that improves patient outcomes. The challenges outlined for implementation of MIGS in pediatric cases have implications for the development of AI-guided surgical procedures that could mitigate obstacles and promote application of these approaches and devices.

## AI opportunities for access to clinical or educational information in glaucoma

A series of articles in this Research Topic identify opportunities for AI approaches to improve patient access to care and information through telemedicine, mobile screening, and outreach in glaucoma.

The original research by Birhanie et al. examined adherence to glaucoma treatments in 189 patients in North West Ethiopia. They concluded that knowledge, distance from the patients’ homes to hospitals, and scheduling problems are significant factors associated with poor adherence to glaucoma treatment.

The original research by Miller et al. reports the incidence of undiagnosed glaucoma in the Jirel ethnic group in Nepal as 94%. The high rate of undiagnosed cases is attributed to the applicability and accessibility of screening tools. This study highlights the significant potential for AI approaches in glaucoma detection using low-cost, portable technologies as well as the necessity to incorporate regional and ethnic diversity in AI algorithms for enhanced generalizability and model performance.

The mini-review by Yadav and Tanwar highlights the impact of the COVID-19 pandemic on glaucoma diagnosis and progression as well as the potential for telemedicine to prevent future impact. Challenges and limitations of teleglaucoma in its current state reveal the need for implementation of AI protocols that can support teleglaucoma screening and management through improved sensitivity and specificity. The gaps in technology and their potential solutions presented here have implications for access to medical care not only in pandemic conditions, but also in underserved and remote populations.

The original research article by Wu et al. investigates the efficacy of ChatGPT as a tool for patient education in glaucoma. Their findings revealed that ChatGPT is more effective for generic information about glaucoma, but is currently limited in its ability to provide detailed and directed information. The authors suggest that the use of ChatGPT in patient education could be improved by intentional tuning of ChatGPT and similar large-language or multimodal foundation models by ophthalmologists and the associated boards and societies.

In a nutshell, this Research Topic emphasizes the importance and challenges of current AI approaches for glaucoma assessment from basic research and clinical translation to healthcare and public education. It also identifies opportunities for critical refinements for AI use in glaucoma to improve generalizability and model performance, while enhancing accessibility and breaking the barriers to benefit rural and underserved communities. Despite the increasing number of glaucoma patients with the aging population, upon better understanding of glaucoma pathology and technological advancements, we are hopeful that the incorporation of fine-tuned AI models for glaucoma applications will facilitate earlier detection and more effective management, which can help reduce the prevalence and socioeconomic burden of this irreversible but preventable blinding disease.

## Author contributions

KC: Writing – original draft, Writing – review & editing. RS: Writing – original draft, Writing – review & editing.

